# Lung-specific RNA interference of coupling factor 6, a novel peptide, attenuates pulmonary arterial hypertension in rats

**DOI:** 10.1186/s12931-016-0409-5

**Published:** 2016-08-04

**Authors:** Jie Yin, Shuling You, Nannan Li, Shouhai Jiao, Hesheng Hu, Mei Xue, Ye Wang, Wenjuan Cheng, Ju Liu, Min Xu, Suhua Yan, Xiaolu Li

**Affiliations:** 1 Department of Cardiology, Shandong Provincial Qianfoshan Hospital, Shandong University, No. 16766 Jingshi Road, Lixia District, Jinan, Shandong Province China; 2 Department of Pathology, Adicon Company, Wangkai Infectious Diseases Hospital of Zaozhuang City, Zaozhuang, Shandong China; 3 Department of Emergency, Shandong Provincial Qianfoshan Hospital, Shandong University of Traditional Chinese Medicine, Jinan, Shandong Province China; 4 Medical Research Center, Shandong Provincial Qianfoshan Hospital, Shandong University, Jinan, Shandong Province China; 5 Department of Emergency, Shandong Provincial Qianfoshan Hospital, Shandong University, Jinan, Shandong Province China

**Keywords:** Pulmonary Hypertension, Coupling factor 6, Prostacyclin, Gene therapy

## Abstract

**Background:**

Pulmonary arterial hypertension (PAH) is a progressive and life-threatening disease associated with high morbidity and mortality rates. However, the exact regulatory mechanism of PAH is unknown. Although coupling factor 6 (CF6) is known to function as a repressor, its role in PAH has not been explored. Here, we investigated the involvement of endogenous CF6 in the development of PAH.

**Methods:**

PAH was induced with monocrotaline (MCT), as demonstrated by significant increases in pulmonary artery pressure and vessel wall thickness. The adeno-associated virus (AAV) carrying CF6 short hairpin RNA (shRNA) or control vector (2×10^10^ gp) was intratracheally transfected into the lungs of rats 2 weeks before or after MCT injection.

**Results:**

A 2-6-fold increase in CF6 was observed in the lungs and circulation of the MCT-injected rats as confirmed by qRT-PCR and ELISA. Immunohistochemistry analysis revealed a small quantity of CF6 localized to endothelial cells (ECs) under physiological conditions spread to surrounding tissues in a paracrine manner in PAH lungs. Notably, CF6 shRNA effectively inhibited CF6 expression, abolished lung macrophage infiltration, reversed endothelial dysfunction and vascular remodeling, and ameliorated the severity of pulmonary hypertension and right ventricular dysfunction at 4 weeks both as a pretreatment and rescue intervention. In addition, the circulating and lung levels of 6-keto-PGF1a, a stable metabolite of prostacyclin, were reversed by CF6 inhibition, suggesting that the effect of CF6 inhibition may partly be mediated through prostacyclin.

**Conclusions:**

CF6 contributes to the pathogenesis of PAH, probably in association with downregulation of prostacyclin. The blockage of CF6 might be applied as a novel therapeutic approach for PAH and PA remodeling.

**Electronic supplementary material:**

The online version of this article (doi:10.1186/s12931-016-0409-5) contains supplementary material, which is available to authorized users.

## Background

Pulmonary arterial hypertension (PAH) is a rare but life-threatening disease characterized by pulmonary vasoconstriction, endothelial cell proliferation, smooth muscle cell proliferation, and *in situ* thrombosis, leading to progressive pulmonary hypertension and ultimately causing right ventricular (RV) failure and death [1–3]. Current therapy with prostacyclin analogs, endothelin-1 receptor blockers, and phosphodiesterase inhibitors improves symptoms and exercise tolerance, but persistent morbidity and mortality indicate that important pathogenic mechanisms are minimally affected. Thus more effective therapeutic approaches are urgently needed [4, 5]. Recently, transfer of the human prostacyclin synthase (PGIS) gene has been shown to ameliorate monocrotaline (MCT)-induced PAH progression in rats [6]. However, the molecular networks regulating prostacyclin are largely unknown. Coupling factor 6 (CF6), a novel essential vascular constrictor, is a known endogenous inhibitor of prostacyclin (PGI_2_) [7].

CF6 is a subunit of the stalk that joins the membrane domains, F1 and Fo (F_1_F_o_ complex) of mitochondrial adenosine triphosphate (ATP) synthase. CF6 is released from the surface of vascular endothelial cells by mechanical forces such as the tumor necrosis factor-alpha (TNF-α), shear stress, and high glucose levels [8, 9]. More recently, studies showed that plasma membrane-bound ATP synthase in the vascular endothelial cells functions as a receptor for CF6 and may play an important role in affecting vascular function by increasing the concentration of intracellular proton, acidosis [10]. CF6 suppresses PGI_2_ synthesis by inhibiting cytosolic phospholipase A [7] as well as the synthesis of nitric oxide (NO) generation via upregulation of asymmetric dimethylarginine [10], thereby profoundly affecting vascular function. Specifically, the CF6 injection consistently induces an elevation in arterial blood pressure [9]. In CF6-overexpressing transgenic mice, endothelial dysfunction increases, and wall thickness and inflammation are increased in small vessels [11]. These data suggests that CF6, which directly inhibits PGI_2_ synthase, may be present in pulmonary arteries and function as a novel target in the treatment of PAH.

Therefore, we aimed to determine the involvement of CF6 in PAH development using MCT-induced PAH rat models. CF6 RNA interference (RNAi) was used to further evaluate the effects of CF6 inhibition on PAH progression. Here, we have demonstrated for the first time that CF6 is released into the extracellular space in MCT-injected rats and that it contributes to PAH development. Our findings provide novel insights into the molecular basis of PAH and present a potential therapeutic target for this disease.

## Methods

### Preparation of AAV2-CF6-shRNA

A U6 promoter-driven shRNA expression system was established in an AAV2 vector. Green fluorescent protein (GFP) expression was separately controlled by a CMV promoter as a marker for transduction efficiency. CF6 shRNA was designed based on the siRNA sequence (GenBank Acc. NM_053602.2) using an siRNA design tool (Biomiao, Beijing, China) and was screened according to the guidelines reported by Elbashir SM et al. [12]. Four selected siRNA target sequences were inserted between the KpnI and EcoRI sites in a U6-CMV-EGFP/AAV vector, and an optimal CF6 target (sequence: 5′-CAGGACTTAAAGGCTCTTAAT-3′) was selected. A recombinant adenovirus carrying an siRNA sequence targeting the eGFP reporter gene (sequence: 5′-CACCGTTCTCCGAACGTGTCACGTCAAGAGATTACGTGACACGTTCGGAGAATTTTTTG-3′) was included as a control. Both the adenovirus-CF6-shRNA and negative control vectors contained the sequence encoding GFP. All constructs were verified by DNA sequencing, all viral vectors were generated by triple-plasmid cotransfection of human 293 cells, and recombinant virions were column purified as previously described [13]. Next, viral titers were determined using qPCR [14]. The resulting AAV2-CF6-shRNA titer was determined to be 2.5 × 10^11^ vector genomes (vg)/ml, and the AAV2-GFP titer was 1 × 10^12^ vg/ml.

### Animal models

Sprague–Dawley rats (6–7 weeks old, weighing 200–250 g, obtained from the Laboratory Animal Center, Chinese Academy of Science, Beijing) were used in this experiment. The rats were housed at 20 ± 3 °C under a 12-h light/12-h dark cycle with free access to food and water. All procedures were carried out according to approved protocols and guidelines established by the Shandong University Institutional Animal Care and Use Committee.

CF6 expression study was performed in 2 models of PAH: I: animals received a single subcutaneous injection of MCT (Sigma, St. Louis, MO) (60 mg/kg), which is a pyrrolizidine alkaloid that has been previously reported to induce PAH at 2 to 3 weeks after injection [15]. The animals in the sham group received subcutaneous injection of saline. The 24 survivors of the 40 enrolled rats (mean survival rate = 60 %) were randomly assigned to the following 4 groups: 1) the MCT-vehicle-1 week group (*n* = 6); 2) MCT-vehicle-2 week group (*n* = 6); 3) MCT-vehicle-3 week group (*n* = 6); and 4) MCT-vehicle-4 week group (*n* = 6). II: animals intubated via tracheotomy, and ventilated with a small-animal ventilator (HX-300S, TME, Chengdu, China), with an adjusted rate of 60 breaths/min and a tidal volume set to 1.1–1.3 ml/100 g body weight, then received left unilateral pneumonectomy as described previously [16]. Seven days after pneumonectomy rats received MCT or saline injection. As a result, 24 survivors of the 56 enrolled rats (mean survival rate = 43 %) were randomly assigned to the 4 groups as reported in I (*n* = 6 per group).

CF6 knockdown experiment was performed to evaluate the prevention and reversal effect of CF6 inhibition, the rats were randomly assigned to the following groups: the sham group (*n* = 20), MCT-vehicle group (*n* = 30), MCT-control group (*n* = 60) and MCT-shRNA group (*n* = 40). The MCT-control group received intratracheally injection of AAV-GFP-shRNA, while the MCT-shRNA group received intratracheally of AAV-shRNA-CF6 (80 μl; 2.5 × 10^11^ vg/ml) at 2 weeks prior or 2 weeks after MCT injection [6]. The MCT-control and MCT-shRNA groups were further divided into two subgroups by virus injection time-point: i.e. -2w-MCT group (*n* = 30), 2w-MCT group (*n* = 30), −2w-shRNA group (*n* = 20), and 2w-shRNA group (*n* = 20). All animals were sacrificed at 4 weeks after MCT injection. The rats were monitored daily until they developed pulmonary hypertension symptoms such as weight loss and tachypnea until 28 days after MCT administration. Hemodynamic, morphologic, and biochemical assessments were performed.

### Echocardiography and hemodynamic measurements

The rats in the experimental groups were anesthetized by intraperitoneal injection of sodium pentobarbital (30 mg/kg). The room temperature was maintained at approximately 25 °C to avoid hypothermia. A Visual Sonics Vevo 770 echocardiographic machine (Visual Sonics, Toronto, Canada) equipped with a 14-MHz linear transducer was used to assess cardiac function. The measurements were performed in a blinded manner by an echocardiographic expert. Short- and long-axis B-dimensional parasternal views of both ventricles at the level of the papillary muscles were acquired to visualize the areas of the left ventricle (LV) and the right ventricle (RV). Cardiac output and stroke volume were obtained from the B-mode long axis according to Simpson's method, while the pulmonary artery diameter and RV wall thickness were obtained in M-mode. Doppler was applied to the pulmonary artery to obtain the pulmonary artery acceleration time [17].

Blood pressure was evaluated by the tail-cuff method, using a non-invasive automatic blood pressure recorder (BP-98A; Softron, Tokyo, Japan). Each value was the average of at least three consecutive data [18]. Prior to sacrifice of the animals, RV systolic pressure (RVSP) was transduced from the right jugular vein into the vena cava, into the right atrium and then into the right ventricle using a 1.4 F Millar Mikro-Tip catheter transducer (Millar Instruments Inc., Houston, TX). The position of the catheter into the right ventricular was vadilated by acutely increased pressure wave accompanied by the loss of resistance, then RVSP was measured with Power Lab monitoring equipment (Millar Instruments). Hemodynamic values were automatically calculated using a LabChart 7.0 physiological data acquisition system (AD Instruments, Sydney, Australia). The animals were then euthanized before being sacrificed.

### Tissue processing and histology

After completion of the above measurements, cardiac arrest was induced by injection of 2 mmol KCl through the catheter. Blood from the RV was stored at room temperature for 1 h and was then centrifuged at 3000 rpm at 4 °C for 15 min. Blood serum was collected and stored at −80 °C, and the rats were euthanized by exsanguination. Next, the lungs were weighed, and the L/BW ratio was calculated. The right lung was removed and frozen in liquid nitrogen for Western blot analysis. The left lung was inflated with 0.5 % low-melting agarose at a constant pressure of 25 cm H_2_O, fixed in 10 % formalin for 24 h and used for small pulmonary artery and IHC analyses. Then, the heart was excised and the weight ratio of the right ventricle to the left ventricle plus the septum (RV/LV + S) was determined using Fulton’s index [19].

### Immunohistochemistry

The left lung lobes were longitudinally cut and processed as described previously [17] by preparing standard formalin-fixed, paraffin-embedded tissues for HE or regular immunohistochemistry staining. Tissue samples were sectioned at a thickness of 5 μm [20]. Sections stained with hematoxylin and eosin (HE) were examined at 400x magnification to determine the severity of PAH in vessels with diameters of either < 50 or 50 to 100 μm, including the percentage with medial hyperplasia and the percentage occluded [21]. Anti-CF6 (1:150, Abcam), α-SMA (1:500; Abcam), PCNA (1:1000; Millipore) and anti-CD68 (1:400; Santa Cruz) antibodies were used as primary antibodies. Subsequently, slides were incubated with an ABC Elite Kit (Vector Laboratories) and DAB substrate (Vector Laboratories) and counterstained with hematoxylin.

Lung samples from 10 patients with NCSCL at the time of a lobectomy or pneumonectomy for localized lung cancer in the Department of Thoracic Surgery (Qianfoshan Hospital of Shandong University) from 2012 to 2016. Human lung specimens was fixed in formalin for at least 72 h, and then paraffinembedded and cut into 5-mm slices for histology processing. Immunohistochemistry was performed as described above, except that the anti-CF6 polyclonal antibody was used at a dilution of 1:300.

A pathologist blinded to the study reviewed 10 sections per lung. All images were obtained using an Olympus LCX100 Imaging System and analyzed with ImageJ software (version 1.38x; National Institutes of Health). The studies were approved by the Institutional Review Board of Shandong University.

### Western blotting

For immunoblot analyses, RIPA buffer (Beyotime Institute of Biotechnology, Jiangsu, China) was used to extract total protein from frozen lung tissues. The quantity of protein extracted from the tissues was measured using a BCA protein assay reagent kit (Pierce). An equal amount of total protein (80 μg of lane) from each sample was resolved on a 5–8 % SDS-PAGE gel and transferred onto a polyvinylidene difluoride (PVDF) membrane. The membranes were blocked with 5 % nonfat dry milk in PBST containing 0.05 % Tween 20 and incubated overnight at 4 °C with a CF6 primary antibody (1:1500; Abcam). The blots were developed using an enhanced chemiluminescence (ECL) detection kit (Millipore) and visualized using a FluroChem E Imager (Protein-Simple, Santa Clara, CA, USA). Measurements to determine the relative densities were normalized to that of a standard protein (GAPDH) (Proteintech, Wuhan, China) using NIH Image J software.

### qRT-PCR

Total RNA was extracted from lung tissues with Trizol reagent (Invitrogen). cDNA was synthesized from 2 μg RNA using a Prime Script RT Reagent Kit (TaKaRa, Dalian, China) according to the manufacturer's instructions. CF6 mRNA expression was determined using gene-specific primers and SYBR Green 1 with a Bio-Rad iQ5 Multicolor Real-Time PCR machine (Bio-Rad Laboratories). For each sample, both GAPDH and the target gene were amplified in triplicate in separate tubes. Relative gene expression was calculated by the 2^-ΔΔCT^ method [22] and normalized to GAPDH expression. The primers used in this study were as follows:

- CF6: forward: 5′-TGTCCTTCGGTCAGCAGTCTC-3′, reverse, 5′-AACTTATCCATCTCTCCTTTA-3′;
- GAPDH: forward, 5′-AGATCCACAACGGATACATT-3′, reverse, 5′-TCCCTCAAGATTGTCAGCAA-3′.

### Determination of endothelial dysfunction in the pulmonary artery

After the rats received MCT for 28 days, the branch of the pulmonary artery next to the heart was removed, and the pulmonary arteries from the individual rats were rapidly separated and placed in cold oxygenated Krebs’ solution composed of the following (mmol/L): 118.3 NaCl, 4.7 KCl, 2.5 CaCl_2_, 1.2 KH_2_PO_4_, 1.2 MgSO_4_-7H_2_O, 25 NaHCO_3_ and 11.1 glucose. The tissues were then oxygenated with carbogen gas at 37.0 ± 0.5 °C. The right and left pulmonary arteries were carefully dissected to remove fat and connective tissue and cut into 3 mm-wide rings. After an equilibration period of 2 h at 1.5 g resting tension, the preparations were exposed to increasing concentrations of Phe (10^−10^ to 10^−5^ mol/L). After a contraction plateau was reached, the pulmonary artery rings were exposed to increasing concentrations of ACh (10^−10^ to 10^−5^ mol/L) to determine the vasodilation capacity and endothelial dysfunction.

### ELISA

The blood samples obtained from the RV were stored at room temperature for 1 h and were then centrifuged at 3000 rpm at 4 °C for 15 min. Serum samples were collected and stored at −80 °C. The level of 6-keto PGF1a, a stable metabolite of prostacyclin, was measured to assess prostacyclin production. The serum 6-keto PGF1a and CF6 levels were measured using a commercial enzyme immunoassay kit (EIA Assay Design, Inc.; Ray Biotech, Norcross, GA, USA) according to the manufacturer's instructions. Rat organs were harvested and homogenized to measure the 6-keto PGF1a levels in the tissues. The results were expressed as picograms of 6-keto-PGF1a per milligram of protein.

### Statistics

The data are presented as the mean ± standard deviation (SD). The unpaired *t*-test was used to compare values between two groups. ANOVA was used to compare differences between more than two groups, followed by a Newman–Keuls test. Analyses were performed using SPSS 17.0 software (SPSS Inc. Chicago, IL, USA). A p-value of < 0.05 was considered statistically significant.

## Results

### Validation of PAH animal model

**Fig. 1:**
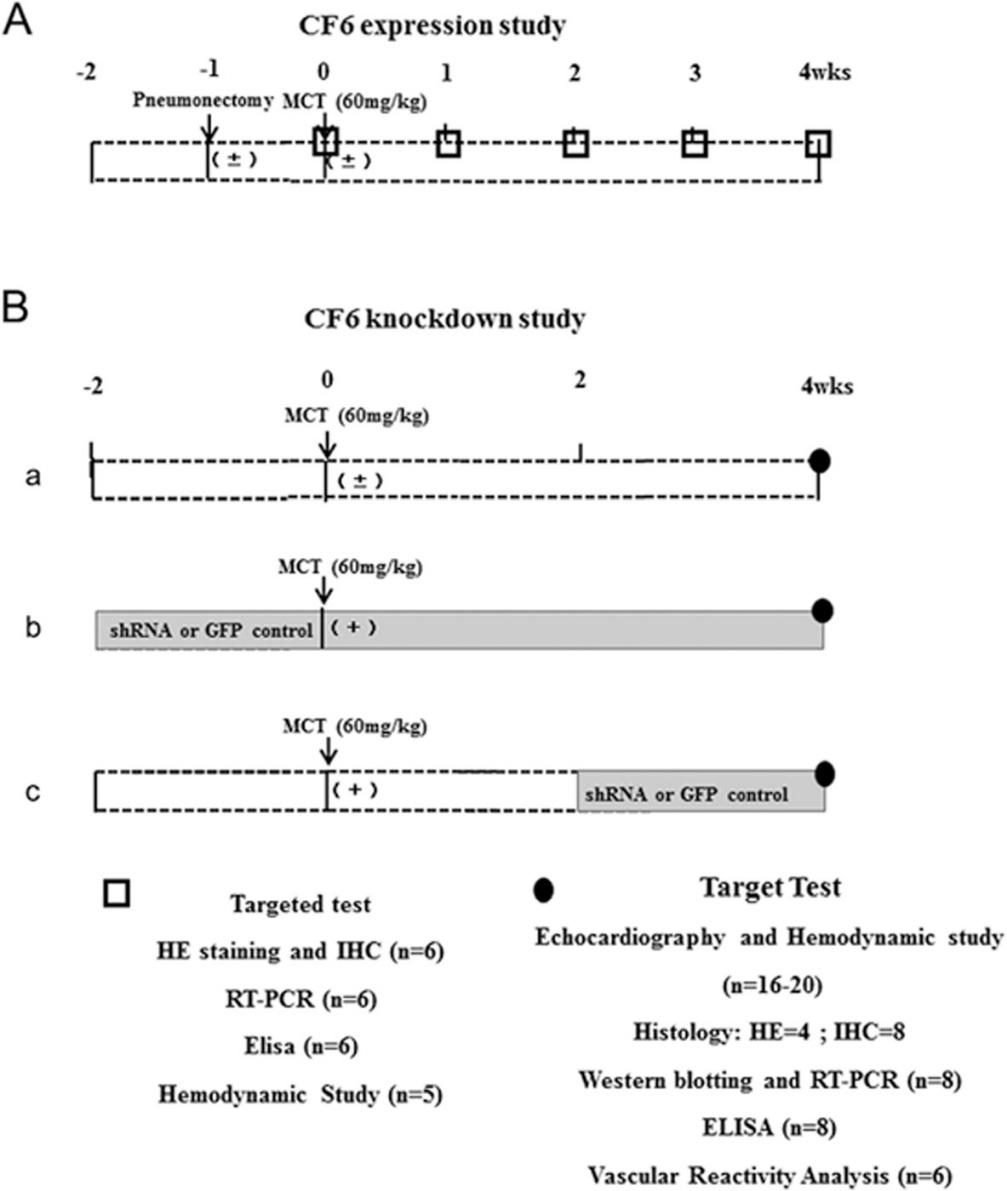
Timeline of the experiment and schematic diagram for (**A**) CF6 expression study. (**B**) CF6 knockdown study: (**a**) sham and MCT-vehicle group; (**b**) MCT-shRNA group and MCT-control group for prevention study; (**c**) MCT-shRNA group and MCT-control group for reversal study

The experimental timeline is shown in Fig. 1. First, we validated the MCT-induced PAH model, the most common experimental model for PAH in rats [23]. Pulmonary hypertension was indicated by a significant increase in RV systolic pressure (RVSP) compared with the control rats (Additional file 1: Figure S1A, *p* < 0.05). Progressive increases in RV/ (LV + S) (Additional file 1: Figure S1B) were also observed. These changes were associated with muscularization and wall thickening of the pulmonary arterioles (Additional file 1: Figure S1C–H). Moreover, pneumonectomized rats that received MCT not only consistently demonstrate neointimal formation (Additional file 1: Figure S2C-H), but also higher RVSP (Additional file 1: Figure S2A) and higher RV/ (LV + S) (Additional file 1: Figure S2B). High death rates were observed among the PAH rats at 4 weeks after MCT injection; however, no control rats died during the experimental period. In the present study, we used the two model to investigate the expression of CF6 in MCT-induced PAH.

### CF6 expression in PAH rats

**Fig. 2:**
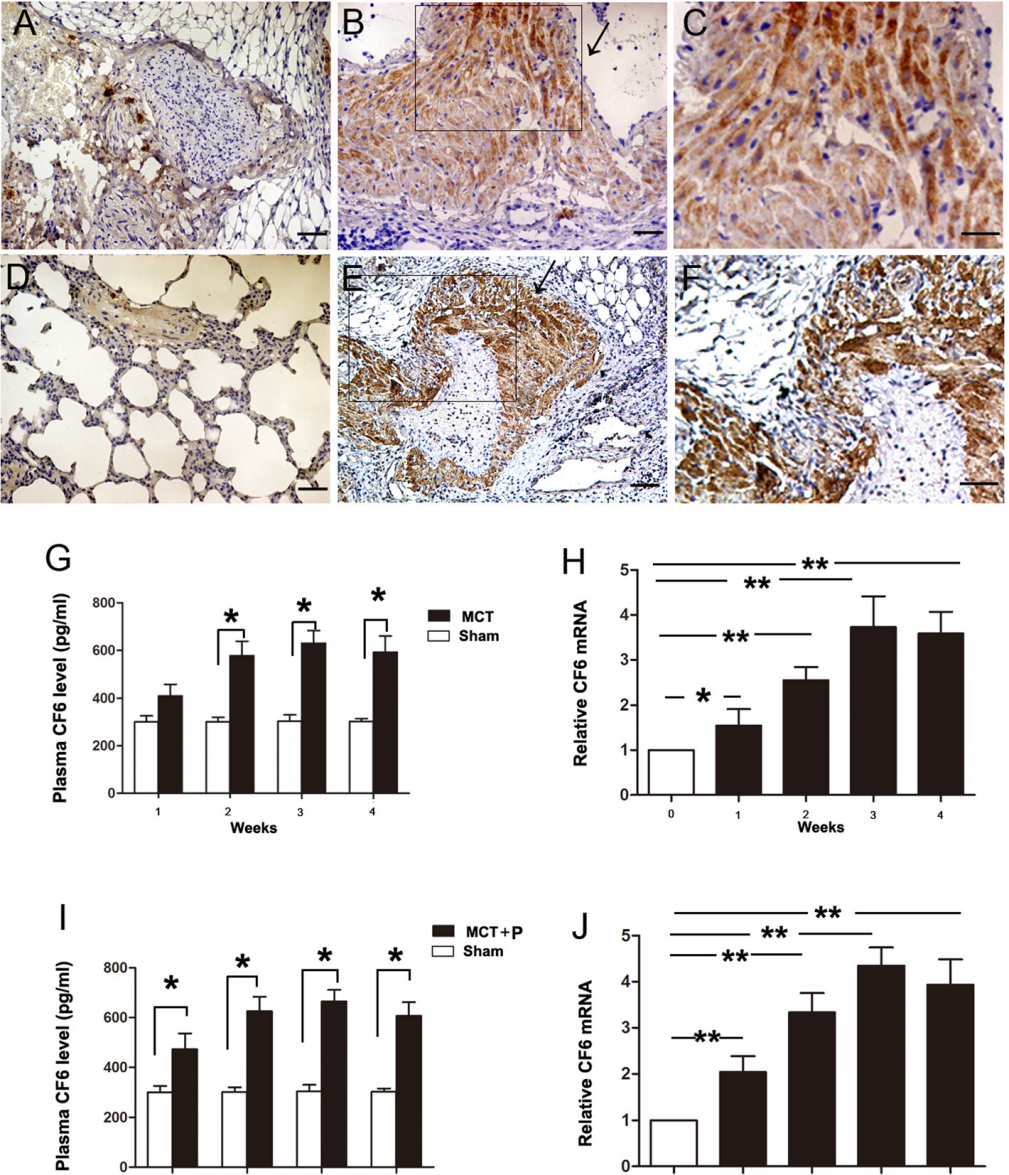
**a**-**f** IHC staining of CF6 in the sham group (**a**, **d**), MCT-vehicle group (**b**, **c**) and MCT plus left pneumonectomy group (**e**, **f**) at 4 w after MCT injection. Quantification of serum CF6 levels at 1 w, 2 w, 3 w, 4 w by ELISA in MCT-vehicle group (**g**) and MCT plus left pneumonectomy model (**i**). Relative CF6 mRNA levels in MCT-vehicle group (**h**) and MCT plus left pneumonectomy model (**j**), as detected by quantitative RT-PCR. The results are expressed as the mean ± SD. Bar = 30 μm. **p < 0.01 and *p < 0.05. IHC, immunohistochemistry; MCT, monocrotaline; PAH, pulmonary artery hypertension; P, pneumonectomy

We first measured the CF6 mRNA level in lung tissue and the circulation in both MCT induced and MCT plus pneumonectomy induced PAH. We found that under physiological conditions, the CF6 mRNA level was low in the lung tissue. However, a 2-6-fold increase was observed in the lungs of the MCT-injected rats in a time-dependent manner, starting at 1 w, peaking at 3w and remaining high at 4 w after MCT injection relative to the levels in the sham group (Fig. 2h, *p* < 0.01). Meanwhile, the circulating CF6 level did not increase until 2 w after MCT injection, and it remained high at 4 w (Fig. 2g). We further reveal the expression profile in MCT plus left pneumonectomy in which the pattern of vascular remodeling resembles the neointimal lesions seen in PAH, to address the problem of determining whether CF6 expression is alerted. As a result, CF6 gene expression and plasma concentration in pneumonectomized MCT- injected rats increased in a similar manner to a higher level than in MCT injected rats (Fig. 2i, j), while the circulating CF6 level increased earlier at 1 w after MCT injection, and it remained high at 4 w (Fig. 2i). To determine CF6 expression in the lung, tissue sections were immunostained for CF6. Immunohistochemistry (IHC) analysis revealed a small quantity of CF6-positive endothelial cells in the pulmonary vasculature (Fig. 2a, d), in consistent with previous findings indicating that CF6 is present on the surface of human vascular endothelial cells. However, in the MCT-injected rats and pneumonectomized MCT- injected rats, intense immunostaining revealed that CF6 was released outside of ECs and spread throughout the arteries and in local tissues in a paracrine manner (Fig. 2b, c, e, f). Given its marked regulation in the PAH rats lung [24], we next sought to examine the expression of CF6 in lung specimens from patients with lung cancer. CF6 was considerably expressed in pulmonary arteries and lung tissues from patients with NSCLC, but was limited expressed in ECs and barely detectable in normal lung tissue (Additional file 1: Figure S3), suggesting that CF6 might be both an obvious marker of impaired endothelium and a novel risk factor contributing to vascular damage. The elevation of CF6 in MCT-induced PAH led us to hypothesize that CF6 might be an initiating factor for PAH, and that blocking CF6 peptide using recombinant AAV-2 carrying the rat CF6 short hairpin small-interfering RNA could prevent development of PAH.

### Intratracheal delivery and AAV2-mediated CF6 knockdown in vivo

**Fig. 3:**
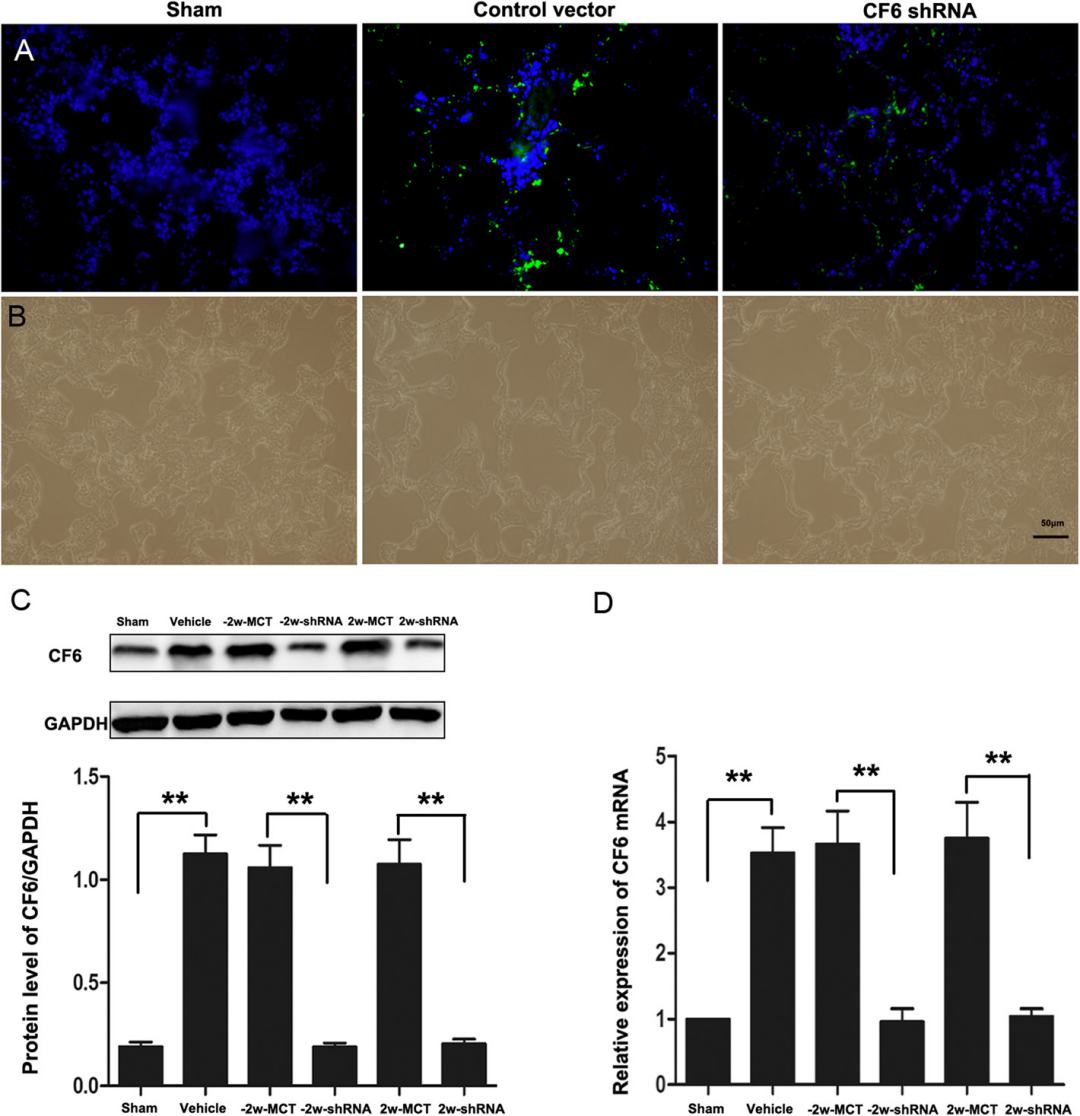
Transduction efficiency after intratracheal injection of CF6 shRNA in vivo. Representative image of GFP expression (**a**) in lung tissues of rats at 2w after CF6 shRNA or GFP vector injection in sham group, control vector group and CF6 shRNA group (*n* = 4 for each group). Cell nuclei were labeled with DAPI DNA stain (blue). Bar = 50 μm. **b**, **c** CF6 (18 KDa) protein levels normalized by GAPDH (36 KDa), as analyzed by Western blot and relative CF6 mRNA levels, as detected by quantitative RT-PCR in the sham, vehicle, −2w-MCT, −2w-shRNA, 2w-MCT and 2w-shRNA groups. **p < 0.01.

As is shown in Fig. 3a, fluorescence microscopic detection of GFP expression in lungs of experimental animals was used to evaluate the distribution of delivered shRNA or control virus. Our results showed that the GFP was expressed in lung tissues, indicating that the shRNA complex could be efficiently delivered to lungs *in vivo* via intratracheal injection (Fig. 3a). RT-PCR and Western blotting were performed to further confirm the knockdown efficiency of CF6 shRNA (Fig. 3b, c). As a result, mRNA and protein levels were clearly lower, in the CF6 shRNA-MCT group compared with the corresponding MCT-control group (*p* < 0.01), while similar to that observed in the normal rats, indicating that CF6 expression was effectively eliminated. Though as a vasoconstrictor, CF6 RNA silencing delivered intratracheally did not affect systemic blood pressure (Table 1). These data suggest that one single-injection of specific shRNA achieved high efficacy for continuous and stable selective deletion of CF6 for at least 4 weeks.

**Table 1:** Hemodynamics and echocardiography data at 28 day after MCT injection

Parameters	Sham	Vehicle	−2w-MCT	−2w-shRNA	2w-MCT	2w-shRNA
No. of surviving rats	20	19	18	18	17	16
Body weight, g	360 ± 8	296 ± 5*	291 ± 6*	335 ± 7*,**	286 ± 7*	323 ± 6*,**
Heart Rate, bpm	418 ± 12	420 ± 16	425 ± 14	422 ± 15	428 ± 16	426 ± 15
Cardiac Output (ml/min)	134 ± 2.3	107 ± 4*	107 ± 4*	116 ± 3.4*	109 ± 4.2*	112 ± 2.8*
Ejection Fraction (%)	66.8 ± 4.2	48.7 ± 4.6*	46.8 ± 3.9*	67.2 ± 5.3**	47.6 ± 4.3*	64.5 ± 3.4**
Pulmonary artery , cm	0.295 ± 0.01	0.41 ± 0.025*	0.38 ± 0.03*	0.317 ± 0.03**	0.42 ± 0.04*	0.336 ± 0.02**
Mean blood pressure	92.5 ± 3.2	88.5 ± 4.7	89.6 ± 3	90.8 ± 2.5	87.4 ± 3.2	88.3 ± 3.6
RV/BW (mg/g)	3.7 ± 0.2	10 ± 0.9*	9.7 ± 1*	5.6 ± 0.8*,**	9.4 ± 0.9*	6.2 ± 0.7*,**
RV wall thickness, cm	0.12 ± 0.01	0.21 ± 0.03*	0.21 ± 0.04*	0.13 ± 0.03**	0.22 ± 0.03*	0.16 ± 0.02**
RV area, mm^2^	11.7 ± 0.3	22 ± 0.7*	21.5 ± 0.6*	12.5 ± 0.5**	21 ± 0.4*	14.6 ± 0.4*,**
LV area, mm^2^	22.1 ± 0.4	22.5 ± 0.5	22.4 ± 0.4	22.3 ± 0.7	22.6 ± 0.5	22.4 ± 0.6

*RV* right ventricle, *LV* left ventricle, *BW* body weight. All values are mean ± SD

*p <0.05 compared with sham group

**p < 0.05 compared with respective control MCT group

### AAV2-mediated CF6 knockdown reduced pulmonary vascular remodeling

**Fig. 4:**
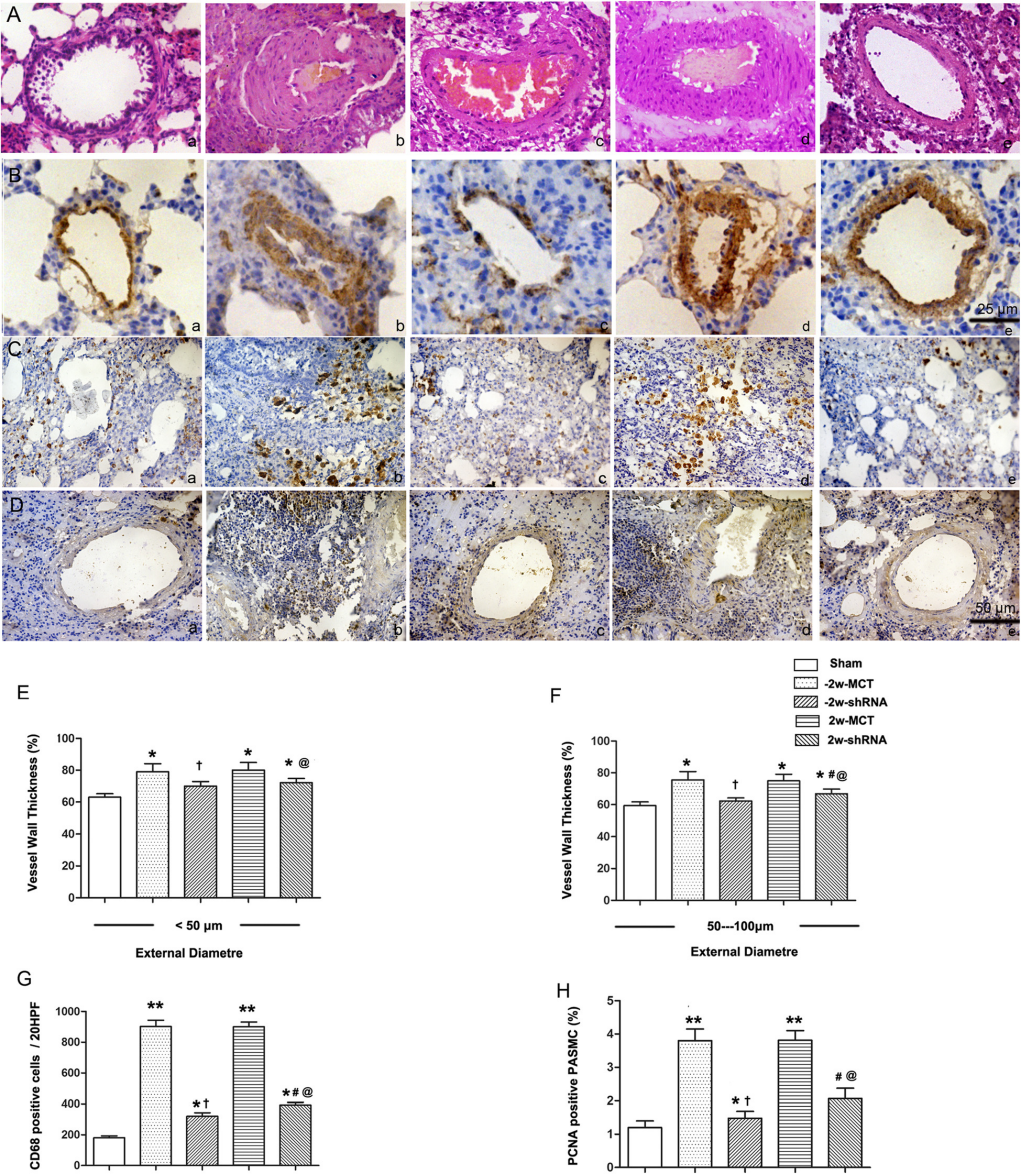
Effect of AAV-CF6 shRNA on pulmonary vascular remodeling. **a** Hematoxylin and eosin and IHC staining of (**b**) alpha-actin with an α-SMA antibody, (**c**) of macrophages with a CD68 antibody, and (**d**) of vascular proliferation of lung tissues with a PCNA antibody in the (**a**) sham, (**b**) -2w-MCT, (**c**) -2w-shRNA, (**d**) 2w-MCT and (**e**) 2w-shRNA groups, respectively. **e** Ratios of vascular medial thickness (i.e., smooth muscle thickness) to the outer diameter (total vessel wall thickness) of the small pulmonary arteries in the PAH and normal rats. **f** Numbers of CD68-positive macrophage cells per 20 high-power fields (HPFs). **g** Relative changes in the number of PCNA-positive cells in pulmonary arterial walls, which were significantly different from the number in the MCT-control group. **p < 0.01, *p < 0.05 compared with sham; †*p* < 0.05 compared with -2w-MCT; #*p* < 0.05 compared with -2w-shRNA; and @ *p* < 0.05 compared with 2w-MCT

PAH causes pulmonary vascular remodeling [15]. Therefore, we evaluated remodeling by measuring wall thickness of the pulmonary arterioles. As shown in Fig. 4, in vessels with diameters ranging from 50 to 100 μm, wall thickness was significantly increased from 63.5 ± 2.1 % (sham group) to 80.2 ± 4.3 % and 79.5 ± 4.56 % (MCT-control groups). Pretreatment with AAV2-CF6-shRNA reduced the IPA wall thickness to 70.3 ± 2.5 % (*p* < 0.05 vs. MCT-control; Fig. 4). Meanwhile, no significant difference in this parameter was found between the MCT-vehicle and MCT-control groups (data not shown). Similar results were observed in vessels with diameters of less than 50 μm. Accordingly, PCNA expression in PASMCs was higher in the control-MCT group compared with the sham group, and CF6 RNAi diminished this increase (*p* < 0.05). The downregulated α-SMA and PCNA expression and reduced intrapulmonary pulmonary artery (IPA) medial wall thickness by CF6 shRNA, implicated that MCT caused decreased severity of pulmonary vascular muscularization, reversed progression of pulmonary vascular remodeling. In addition, densitometric quantification of CD68 protein indicated significantly reduced macrophage infiltration both by treatment and pretreatment with CF6 shRNA (Fig. 4g). These results indicate that CF6 upregulation is involved in MCT-induced pulmonary vascular remodeling.

### AAV2-mediated CF6 knockdown ameliorated pulmonary hypertension

**Fig. 5:**
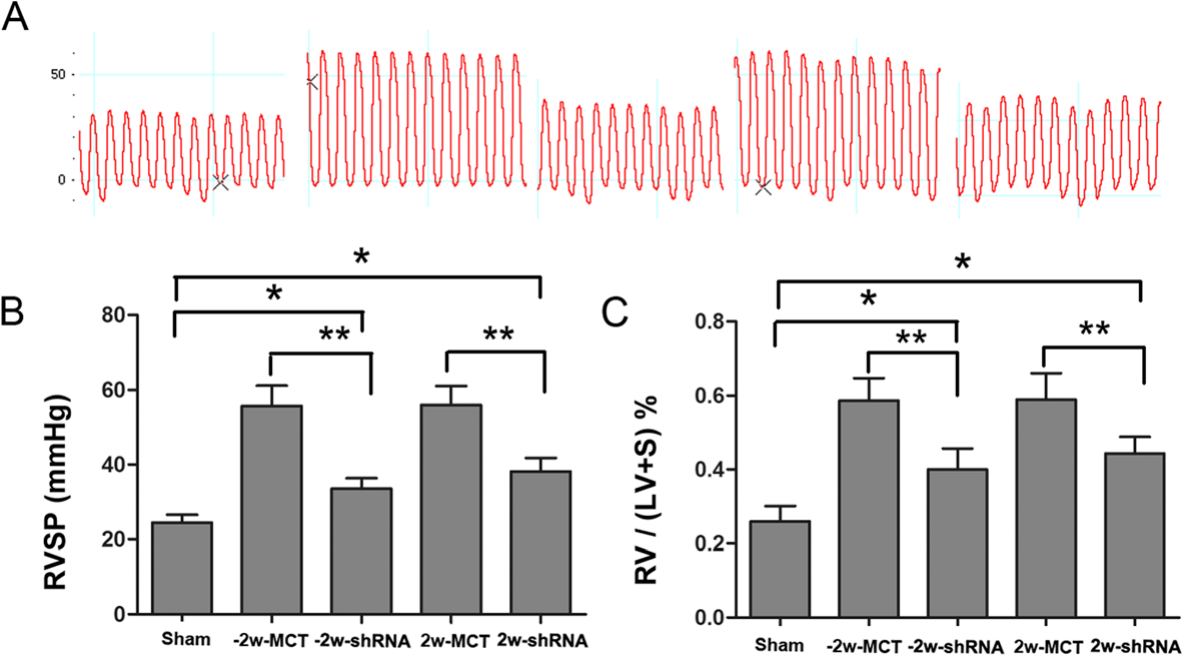
Targeted CF6 silencing alleviates hemodynamic changes and right ventricular hypertrophy in PAH rats at 4 weeks after MCT exposure. **a** Representative hemodynamic data (RVSP) obtained with a Labchart acquisition system. **b** CF6 shRNA decreased RVSP in the MCT-control group. **c** CF6 shRNA also reduced the RV/LV + S ratio in the MCT-treated rats. The data represent the mean ± SD. **p < 0.01 and *p < 0.05. RVSP, right ventricle systolic pressure; RV/LV + S ratio, weight ratio of the right ventricle to the left ventricle plus the septum

We then analyzed the hemodynamic data and echocardiographic changes at 4 w to determine whether CF6 silencing affects right ventricular pressure and right heart hypertrophy. As a result, RVSP was found to be attenuated in the rats pretreated with CF6 shRNA (33.6 ± 2.9 mmHg, *p* < 0.05 vs. the MCT group) (Fig. 5a, b). Similarly, CF6 shRNA administration reduced the increases in the RV/ (LV + S) ratio (*p* < 0.05; Fig. 5c) and the RV/BW (*p* < 0.05; Table 1). Administration of CF6 RNAi 2 weeks after MCT injection completely inhibited the progression of PAH and RV dysregulation as well. No significant differences were found between the two control-MCT groups. These findings indicate that the enhanced CF6 plays an important role in the pathogenesis of MCT-induced PAH and RV dysfunction, and provide a potential therapeutic target.

As shown in Table 1, the rats that received the CF6 shRNA treatment prior to or after MCT administration showed significant reductions in RV wall thickness, RV area, and pulmonary artery diameter compared with the MCT-induced rats; the CO level was slightly but not significantly increased in these animals relative to the controls (*p* > 0.05) due to enhanced stroke volume, but the heart rate was not altered. Furthermore, the LV area in the MCT-injected rats did not significantly change compared with the sham rats. In addition, low death rate was observed in the CF6 shRNA-treated PAH rats. These findings demonstrate that inhibition of CF6 decreases RVSP and RVHI. Our data also demonstrated that treatment with CF6 shRNA improved survival assessed at day 28 compared with MCT alone compared with those transfected with control vector alone both in pretreatment and treatment manner (log rank test *p* < 0.05; Additional file 1: Figure S4A, B). No definite adverse effects were detected after transfer of CF6 shRNA.

### Delivery of CF6 shRNA reversed endothelial dysfunction of pulmonary artery rings in MCT-induced PAH

**Fig. 6:**
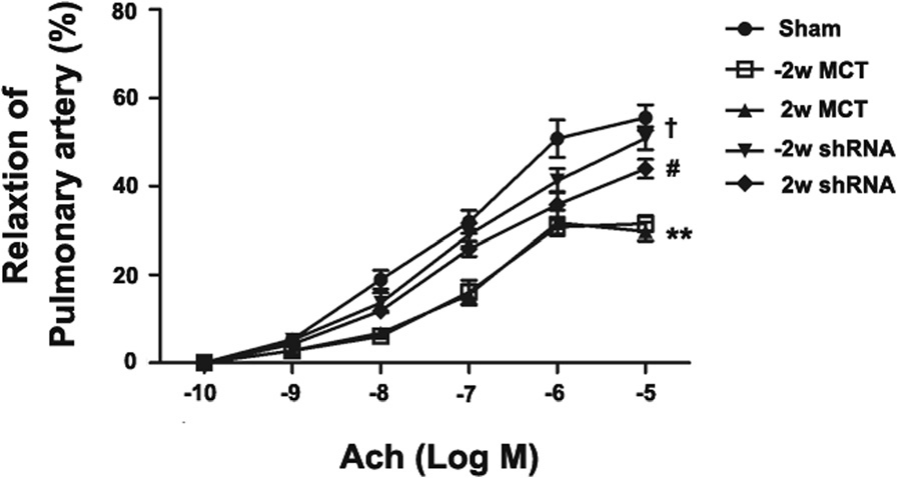
Acetylcholine (ACh)-induced relaxation of pulmonary artery rings from the sham, −2 w-MCT, 2 w-MCT, −2 w shRNA and 2 w shRNA rats. The data are presented as the mean ± SD. **p < 0.01 compared with the sham group; † *p* < 0.05 compared with the −2 w-MCT group; and # *p* < 0.05 compared with the 2 w-MCT group. All groups were evaluated at 28 days after MCT injection

Following completion of the treatment, the pulmonary artery rings were removed from the rats and prepared for isometric tension recording to evaluate endothelial dysfunction. The vasodilator responses of the pulmonary artery rings to increasing concentrations of ACh were reduced in the rats that received MCT. The maximum relaxation induced by ACh was reduced from 55.6 ± 5.1 % (sham) to 31.5 ± 3.0 % and 28.6 ± 3.9 % (MCT-vehicle) (*p* < 0.05 vs. sham). These results indicate that MCT administration induces endothelial dysfunction of the pulmonary artery rings. Intratracheal injection of shRNA CF6 before or after MCT injection restored the maximal relaxation induced by ACh to 50.9 ± 4.5 % and 45.8 ± 6.4 % (*p* < 0.05 vs. MCT-control, Fig. 6), suggesting that CF6 inhibition exerts its beneficial effect by reducing endothelial dysfunction of the pulmonary artery rings in PAH rats.

### Effects of endogenous CF6 on prostacyclin production in lung tissues and serum

**Fig. 7:**
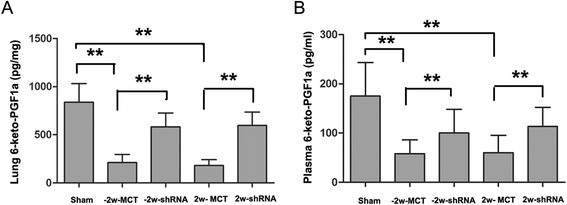
Levels of 6-keto-PGF1a in lung tissue (**a**) and plasma (**b**). The results are expressed as the mean ± SD. **p < 0.01

PGI_2_ plays an important role as an endogenous regulator of vascular homeostasis, inhibiting platelet aggregation and VSMC proliferation and migration [25]. 6-keto-PGF1a, a stable metabolite of prostacyclin, were analyzed to assess prostacyclin production. As illustrated in Fig. 7, the 6-keto-PGF1a production rates at baseline were 175 ± 61 pg/μg and 838 ± 174 pg/ml in lung tissues and serum samples, respectively. MCT injection significantly decreased these rates (both *p* < 0.05), which is negatively correlated with the increased CF6 level. Meanwhile, knockdown of CF6 peptide prevented the MCT induced increase in CF6 and reversed 6-keto-PGF1a production in both the lung tissues (58 ± 24.8 vs. 115 ± 43 pg/μg, *p* < 0.05 and 60 ± 26.8 vs. 91.6 ± 32.9 pg/μg, *p* < 0.05, respectively) and serum samples (180.3 ± 54 vs. 580.6 ± 130.3 pg/ml, *p* < 0.05 and 180.3 ± 54.4 vs. 541.7 ± 111.3 pg/ml, *p* < 0.05, respectively) compared with the MCT-control group. No significant differences were found between the two MCT-control groups. This suggests that the enhanced CF6 may function via inhibition of prostacyclin generation in the progression of PAH.

## Discussion

To our knowledge, the current study is the first to elucidate the role of CF6 in the pathology of PAH. A single injection of MCT caused persistent severe PAH, which shares important features with human diseases, such as pulmonary arterial endothelial cell (EC) injury and subsequent pulmonary artery smooth muscle cell (PASMC) hypertrophy [26]. Based on the model, we demonstrated that (1) CF6 was significantly upregulated in lungs and serum of PAH rats, specifically, in the lung tissues; (2) CF6 gene silencing using AAV2-CF6-shRNA induced down-regulation of CF6 protein in the lung tissues of the PAH rats, along with reversed prostacyclin synthesis; and (3) transfer of CF6 shRNA ameliorated MCT-induced pulmonary vascular remodeling and PAH progression. Finally, we demonstrated that (4) CF6 knockdown as a therapeutic approach in the MCT rats without causing definite adverse effects.

CF6, a component of the peripheral stalk in ATP synthase under physiological conditions, is known to elicit its novel function outside the cells after being released from the plasma-membrane of ATP synthase in a pathological state [27]. In clinical settings, we and others have previously shown that the plasma CF6 level increases in various cardiovascular disorders, where a cause–result connection between increased plasma CF6 and decreased PGI2 has been indicated [8, 28–30]. However, the CF6 level in PAH has not been explored. There exhibited significant overproduction of CF6 in the lung tissues and elevation in circulating CF6 among PAH rats of MCT models and MCT plus pneumonectomy models, most likely due to mechanical stress secondary to the elevated pulmonary pressure and the inflammation activation induced by MCT, such as NF-kB activation and TNF-α releasing [7, 31, 32]. ECs represents a main source of circulating CF6 [9]. In hypertension study, intravenous injection of recombinant CF6 mimetics increased blood pressure transiently, apparently by suppressing PGI_2_ synthesis, whereas a specific antibody against CF6 decreased systemic blood pressure with a concomitant increase in plasma PGI_2_ in hypertension rats. Except for endocrine manner, our data showed that the limited distributed CF6 observed in ECs in normal rats was released into surrounding tissues and may interact with the interact with plasma membrane ATP synthase, the receptor of CF6 presented on vascular cells such as ECs and vascular smooth muscle cells (VSMCs) function in a paracrine manner, supported by a previous study identified that the high local CF6 levels in tissues such as resistance arterioles could directly enhancing Ca^2+^ signaling in VSMCs and vasoconstriction in the mesenteric arteriolar network in a paracrine manner [10]. In addition, the CF6 level did not affect systematic blood pressure in PAH rats. Therefore, it appears that different from the direct role in pathological hypertensive as a systemic hormone and vasoconstrictor, CF6 may function on PAECs and PASMCs in a paracrine manner in PAH, which is consistent with the fact that pulmonary circulation is selectively inhibited in humans with PAH.

We chose the RNAi strategy over CF6 antibodies to further determine whether the high CF6 level is a cause or merely an effect of PAH given that a therapeutic antibody should decrease the circulating levels of free CF6 to zero and have a limited duration of effect [33], and especially considering that CF6 signaling proteins have short half-lives. Various “RNA interference (RNAi) therapeutics” have already entered clinic trials [34]. Investigators showed that AAV-2 is an efficient vector for delivering genes to pulmonary vessels and lungs to treat PAH [35]. We adopted the intratracheal route for AAV2 delivery of CF6 shRNA because the majority of drugs targeting the vasculature will, if administered systemically, affect normal circulation as well; in contrast, gene transfer of specific peptides to bronchial epithelial cells and alveolar cells selectively affects the pulmonary artery in animal models [36, 37]. As a result, CF6 RNAi reversed ECs dysfunction and PA remodeling, indicating that through the secretion of CF6, ECs regulates the proliferation of SMCs in their vicinity. The data described above revealed that, in addition to its vasoconstrictive properties, CF6 is also involved in the proliferation of PASMCs. The downstream signaling of CF6 is mediated via plasma membrane ATP synthase receptor. After combination, the molecular rotary motor, F1-ATPase in ECs and VSMCs, forcefully hydrolyzes ATP in a mode that is in the reverse direction of the mode used in the mitochondria. Then, this hydrolysis inversely rotates the Fo motor against the original clockwise direction, resulting in proton import. CF6 and the plasma membrane-F1Fo complex mediated VSMC remodeling is well established and that the downstream the intracellular pH (pHi) changes are associated with cell proliferation and migration in VSMCs [28]. Additional methods of CF6 intervening in vitro are needed to define the underlying post receptor signaling mechanisms and the direct role of CF6 on ECs and PASMCs.

Suppressed PGI_2_ is a well-known target of the increasing intracellular acidosis of CF6. In PAH, the primary effects of IP receptor activation (e.g. by prostacyclin or its analogues) are the induction of pulmonary artery dilation and the inhibition of vascular smooth muscle cell proliferation. Treatments that target the prostacyclin pathway are crucial for the effective management of patients with PAH [25, 28, 38, 39]. Our results indicated that the impaired PGI_2_ generation was enhanced in PAH by the overproduction of CF6 and restored by CF6 silencing. Therefore, we speculate that the impaired pulmonary vascular PGI_2_ generation may be the target of CF6 and contributes to pulmonary vasoconstriction and the excessive medial hypertrophy observed in this PAH model. In addition, the CF6 peptide may influence PAH by a more direct mechanism that may be independent of the complex effects of PGI_2_. After binding to the β-subunit of the ATP synthase at the cell surface, CF6 could further activate c-Src or RAC1 [7, 10, 40], which potentially enhances Ca^2+^ influx and sensitization [10, 28, 41, 42]. Aberrant expression of these downstream signaling proteins cause a mitochondrial metabolic abnormality that plays a pathophysiological role in PAH. Moreover, RNAi of CF6 effectively prevented macrophage infiltration, which is involved in the development of PAH-associated vascular lesions and has been well documented in humans [43, 44]. This suggests that CF6 plays a critical role in the inflammation process of PAH; however, the complex mechanism remains poorly understood. These theories provide a strong impetus for ongoing efforts to define the mechanisms of CF6.

### Prospective

Although many vasodilators have antiproliferative effects on VSMCs, there is no definitive evidence that pulmonary vascular remodeling in human PH is reversible. Therefore, novel approaches that directly target pulmonary vessel wall pathology are needed to reverse the established pulmonary vascular pathology in PH patients. We found that CF6 levels increased in MCT model, MCT plus left pneumonectomy model and human lung cancer tissue, while the AAV2-based RNAi targeting CF6 reversed the PA remodeling and PH in MCT-induced PAH. The above makes CF6 a target molecule for novel therapeutic strategies in the treatment of clinical PH regardless of the different clinical forms. Future experiments on hypertrophied pulmonary arterioles and lung tissues from human samples with PAH are needed to confirm CF6 expression. Besides, despite of the fact that CF6 is implicated in multiple clinical settings and experimental models, no pharmacologically active small molecule inhibitors of CF6 has been developed. Therefore, the specific plans aiming at blocking CF6 pathway that can be applied in PAH treatment is warranted to be explored in the future.

### Limitations

First, the MCT-induced PAH model does not accurately reflect the clinical picture of PAH in humans and the high death rate could cause potential selective bias. Further investigations of other animal models of PAH (Sugen-hypoxia) are needed to determine the clinical efficacy of CF6 inhibition. Second, our study was performed within a limited window of time and involved only short-term animal research; therefore, the results only provide an impetus for further clinical investigation. Third, the AAV vectors used were capable of inserting their genome at random positions in the host chromosomes, which eventually restricts gene function and may cause mutations. Moreover, the interpretation of RNAi data is complicated by unintended interactions between silencing molecules and cellular components, and additional shRNA sequences should be designed to decrease the risk of unwanted artifactual off-target effects [45]. Despite these limitations, our data demonstrate a potential role of airway delivery of shRNA-CF6 in paracrine inhibition of CF6 secretion into the pulmonary vasculature in PAH human clinical trials.

## Conclusions

Our results demonstrate that CF6 is activated during pathogenesis of PAH. Additionally, targeted CF6 silencing diminishes the MCT-mediated induction of PAH with down-regulation of PGI_2_, decrease in RVSP, reversal of pulmonary arterial remodeling, and improved RV dysfunction. Therefore, CF6 holds a promise therapeutic target for PAH. Our study was time-limited as a short term animal research, and the results only provided us an impetus for further clinical investigations.

## Additional file

Additional file 1
**Figure S1.** Validation of the MCT-induced PAH model. **Figure S2.** Validation of the MCT plus - pneumonectomy induced PAH model. **Figure S3.** CF6 upregulation in human lung cancer tissue. **Figure S4.** Kaplan-Meier survival curves. (DOCX 4583 kb)
