# Supplementary material for: Lung-specific RNA interference of coupling factor 6, a novel peptide, attenuates pulmonary arterial hypertension in rats
Source: Respir Res. 2016 Aug 4;17:99. doi: 10.1186/s12931-016-0409-5 (PMC4973057; doi:10.1186/s12931-016-0409-5)
Supplement: Additional file 1 — Figure S1. Validation of the MCT-induced PAH model. Figure S2. Validation of the MCT plus - pneumonectomy induced PAH model. Figure S3. CF6 upregulation in human lung cancer tissue. Figure S4. Kaplan-Meier survival curves. (DOCX 4583 kb) [file 12931_2016_409_MOESM1_ESM.docx]

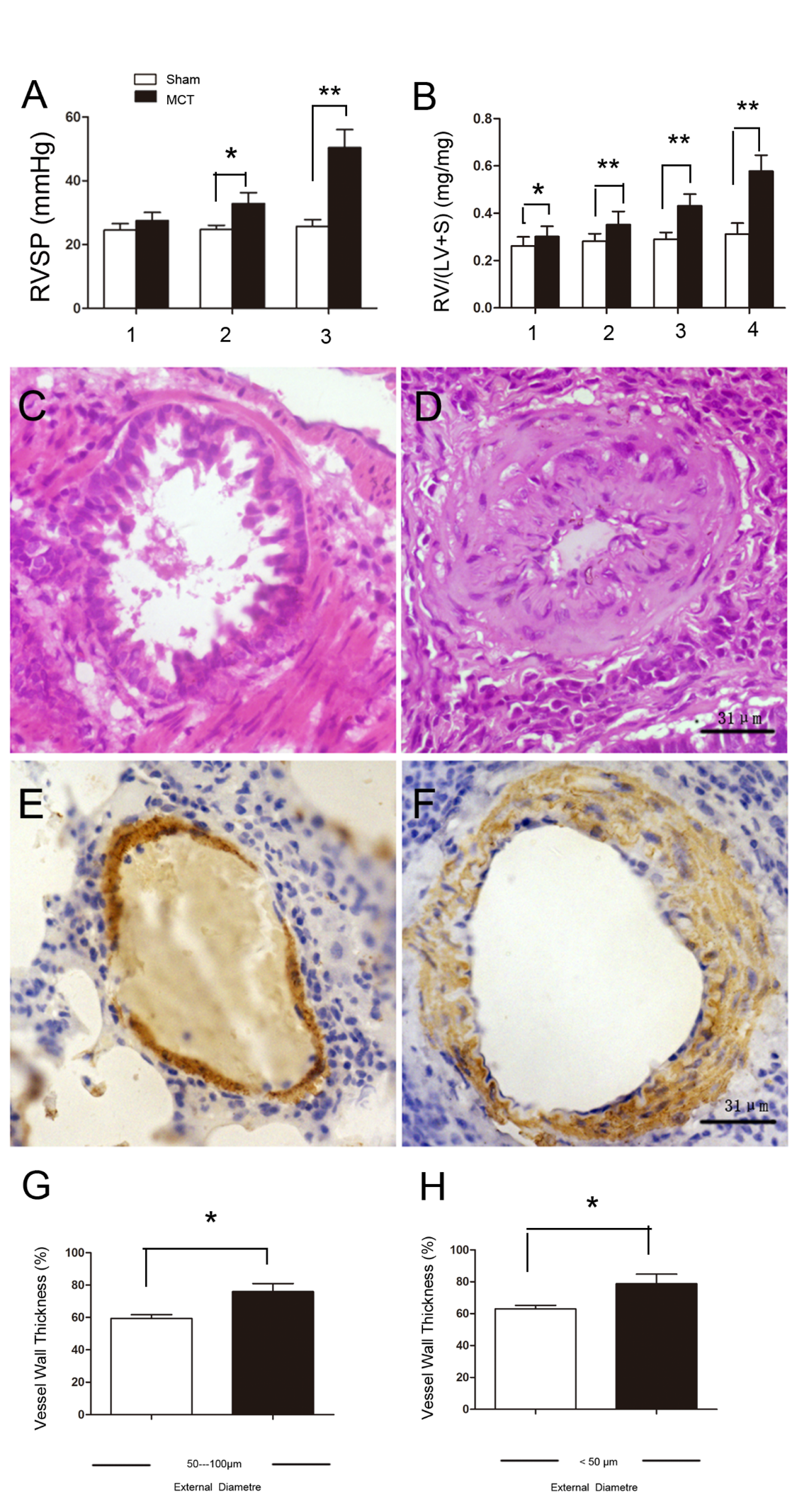


**Figure S1 Validation of the MCT-induced PAH model.** (A–B) Rats were given a single intraperitoneal injection of 60 mg/kg MCT or vehicle, and RV systolic pressure (A) and RV weight (B) were measured 1, 2, 3, or 4 weeks after MCT challenge. (C–F) H&E staining and a-SMA staining of lung tissue sections at the indicated time points after MCT injection. (G, H) The % medial wall thickness was calculated as [(medial thickness×2)/external diameter]×100. Scale bars = 25 µm. n = 6–8 per group. All data are expressed as mean ± SD. **p<0.01 and *p<0.05.


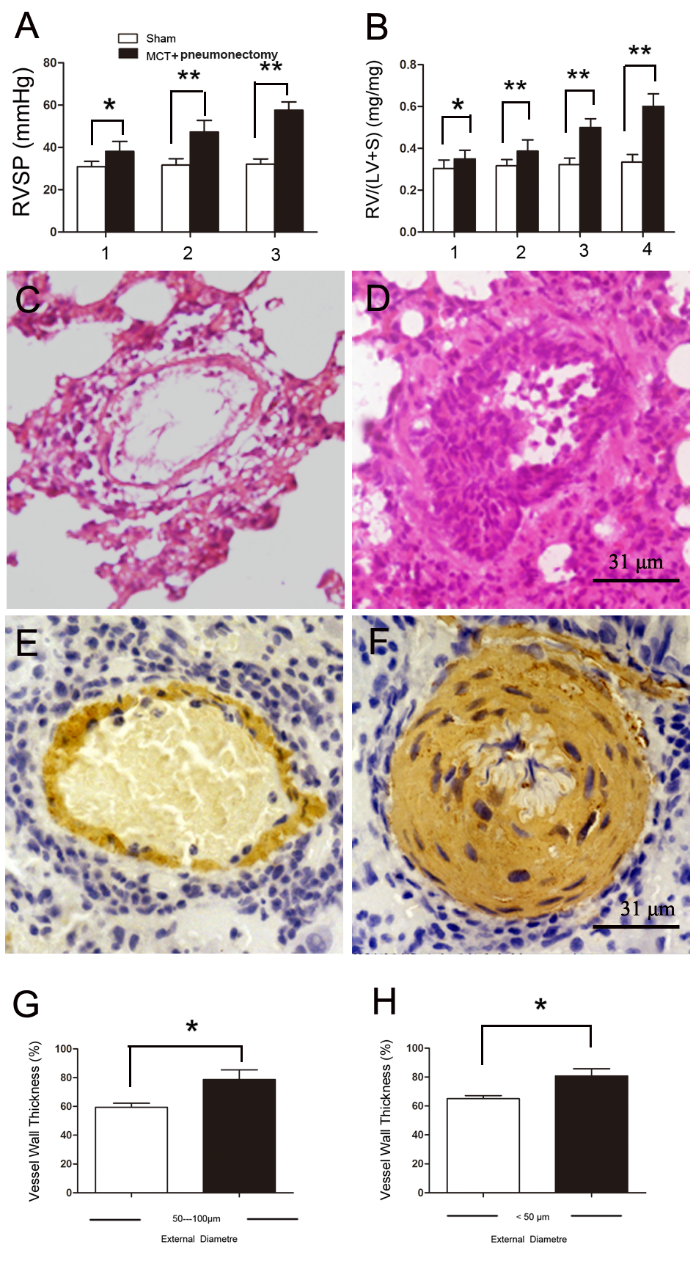


**Figure S2 Validation of the MCT plus - pneumonectomy induced PAH model.** (A–B) Rats were given a single intraperitoneal injection of 60 mg/kg MCT one week after left pneumonectomy or vehicle, and RV systolic pressure (A) and RV weight (B) were measured 1, 2, 3, or 4 weeks after MCT challenge. (C–F) H&E staining and a-SMA staining of lung tissue sections at the indicated time points after MCT injection. (G, H) The % medial wall thickness was calculated as [(medial thickness×2)/external diameter]×100. Scale bars = 25 µm. All data are expressed as mean ± SD. n = 6 per group. **p<0.01 and *p<0.05.

**
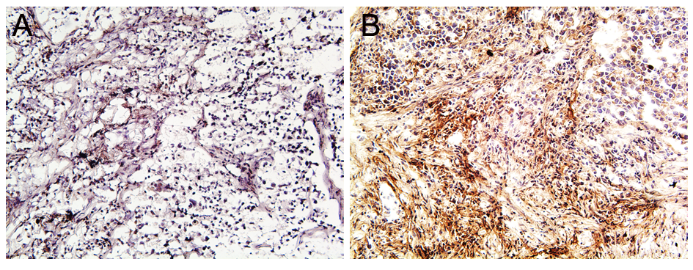
**

**Figure S3 CF6 upregulation in human lung cancer tissue.** (A) Limited amount of positive CF6 immunostaining in ECs in normal lung tissue (at a distance from tumor areas); (B) Strongly positive CF6 immunostaining in NSCLC lung specimens.


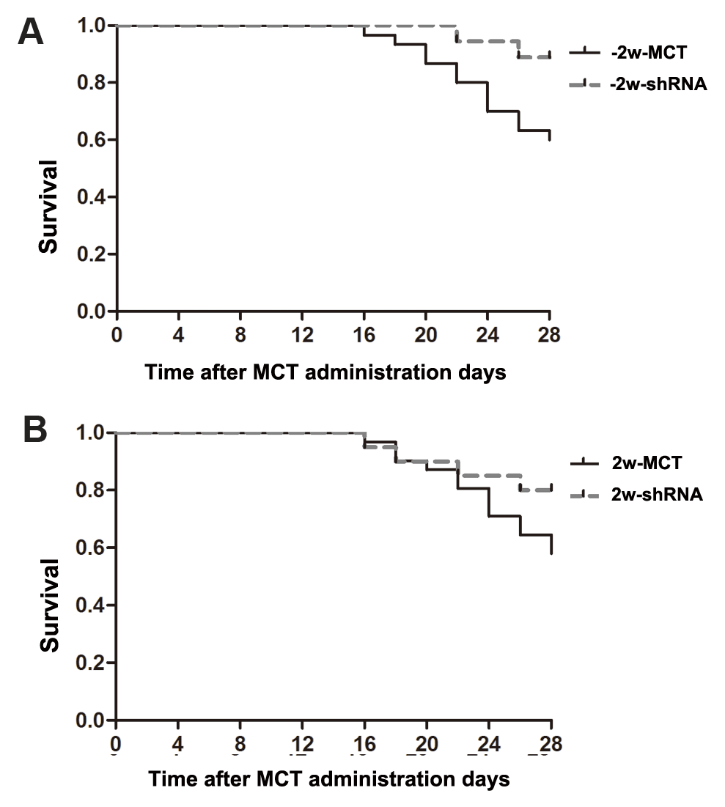


**Figure S4 Kaplan-Meier survival curves.** Kaplan-Meier survival curves showing that MCT rats receiving CF6 RNAi have a significantly higher survival rate than those treated with control vector at 2 weeks prior to (A) and after (B) MCT injection (long-rank test, p<0.05).
